# Pooled analysis of LAMP assay for the diagnosis of norovirus infection

**DOI:** 10.1002/jcla.23919

**Published:** 2021-07-31

**Authors:** Xi‐Feng Qian, Ai‐Ling Duan, Rong‐Xian Huang, Nan‐Xi Li, Ya‐Ping Li, Xu‐Guang Guo

**Affiliations:** ^1^ Department of Clinical Laboratory Medicine The Third Affiliated Hospital of Guangzhou Medical University Guangzhou China; ^2^ Department of Clinical Medicine The Sixth Clinical School of Guangzhou Medical University Guangzhou China; ^3^ Department of Mental Medicine The Mental Health School of Guangzhou Medical University Guangzhou China; ^4^ Department of Clinical Medicine The Second Clinical School of Guangzhou Medical University Guangzhou China; ^5^ Department of Clinical Medicine The Third Clinical School of Guangzhou Medical University Guangzhou China; ^6^ Key Laboratory for Major Obstetric Diseases of Guangdong Province The Third Affiliated Hospital of Guangzhou Medical University Guangzhou China; ^7^ Key Laboratory of Reproduction and Genetics of Guangdong Higher Education Institutes The Third Affiliated Hospital of Guangzhou Medical University Guangzhou China

**Keywords:** high diagnostic accuracy, LAMP, norovirus, RT‐PCR, systematic review

## Abstract

**Background:**

Rapid laboratory detection is essential to diagnose norovirus infection. LAMP has many advantages compared with RT‐PCR for detecting norovirus, including high sensitivity, high specificity, rapidity, low cost, and intuitive results, which can be easily read with the naked eye with the help of color‐based reporters. In this study, we intend to analyze the accuracy of LAMP methods for the diagnosis of norovirus infection.

**Methods:**

Two researchers independently retrieved relevant literature up to January 2021 (PubMed, Web of Science, Cochrane Library, Embase, CNKI, Wan Fang, and VIP). The researchers screened all articles and extracted their research data for meta‐analysis. QUADAS‐2 tool was used to evaluate the quality of the included studies by Review Manager 5.3. Forest plots were performed by Meta‐DiSc 1.4 to evaluate the accuracy of the test. Deeks’ funnel plot symmetry tests were conducted by Stata 15.0 to check the potential publication bias.

**Results:**

Eleven sets of data extracted from the eight included studies were included for meta‐analysis. For the detection of norovirus, the pooled sensitivity, specificity, positive LR, negative LR, diagnostic OR, and their 95% CI were 0.96 (0.95–0.97), 0.99 (0.99–1.00), 91.14 (31.88–260.56), 0.06 (0.04–0.09), and 1473.68 (562.96–3857.70), respectively. Besides, AUC in the SROC curve was 0.9920.

**Conclusion:**

LAMP had high sensitivity and specificity in terms of the diagnosis of norovirus infection. However, further extension of this approach should be researched to ensure the accuracy and practicability of this hopeful test in the future.

## Graphical abstract

The AUC was 0.9920, and Q* was 0.9636 in SROC; the curve was close to the top left corner. The results proved a higher value, suggesting a greater diagnostic potential of LAMP for norovirus infection. One may conclude that the capacity of LAMP in the early diagnosis of norovirus is considerably high. LAMP may be a practical and reliable method for detecting norovirus infection.

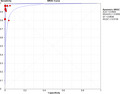

## 1 INTRODUCTION

According to the data in the Centers for Disease Control and Prevention (CDC) and World Health Organization (WHO), norovirus is the dominant cause of acute gastroenteritis that led to diarrhea and vomiting worldwide in recent years.^1^, ^2^ Norovirus causes an estimated 685 million cases and 50,000 child deaths, and costs about $60 billion worldwide due to healthcare costs and lost productivity every year.^1^ Diarrheal diseases were the eighth leading cause of death at all ages and the fifth leading cause of death in children younger than 5 years.^3^ WHO estimated that norovirus was responsible for approximately 1/5 of the cases of diarrheal diseases, leading 35, 000 deaths worldwide in 2010 and constituting the most deaths among the diarrheal diseases.^2^, ^4^, ^5^ Norovirus was divided into at least 6 genogroups (GⅠ–GⅥ), and GⅠ and GⅡ were the predominant genogroups that caused acute gastroenteritis in humans.^6^, ^7^, ^8^ Norovirus infection usually caused a self‐limiting illness in healthy adults with 2‐ to 3‐day course, while in the elderly, young children, and immunocompromised individuals, it was associated with severe complications such as developing severe disease and mortality.^6^, ^7^ Human norovirus has a substantially high prevalence and mortality across both healthcare and community settings, particularly in developing countries, and brings humans a severe global burden of disease.^5^, ^9^, ^10^

Since the rapid spread of norovirus is a major public health issue, rapid laboratory diagnosis is essential to facilitate the execution of appropriate control measures to reduce transmission and outbreaks of the virus. The methods for the detection of norovirus can be concluded in terms of electron microscopy, immunology, and molecular biology.^8^, ^11^ Unfortunately, electron microscopy was not widely available for detecting norovirus in microbiology laboratories because of its low sensitivity, specificity, and expensiveness.^11^ The development of enzyme immunoassays, such as IDEIA and RIDASCREEN, has been challenging because norovirus genotypes that formed most virus antigens were many (*n* = 29) and had the antigenic drift in certain strains.^8^ In addition, due to the problem of sample processing, the IDEIA norovirus assay was at low sensitivity and raised serious questions regarding its usefulness in routine screening for norovirus.^12^ Currently, reverse transcription‐polymerase chain reaction (RT‐PCR) is known as the gold standard in the detection of norovirus due to its high sensitivity and specificity in clinical and environmental samples.^6^, ^8^ However, the disadvantages of RT‐PCR–based assays are also apparent, including high cost, time‐consuming, and dependence on sophisticated equipment and expertise, leading to the limitation in its use in resource‐poor settings without equipped laboratories.^11^, ^13^Loop‐mediated isothermal amplification (LAMP), created by Notomi et al.,^14^ was a remarkable alternative to RT‐PCR for the diagnosis of norovirus infection. LAMP allowed RNA detection in an isothermal condition by using Bst DNA Polymerase that had high displacement activity, which was performed with simple and single‐temperature incubation sources and thus got rid of the limitation of expensive equipment.^6^, ^13^, ^14^ LAMP has many advantages compared with RT‐PCR, including high sensitivity, high specificity, rapidity, low cost, and intuitive results, which can be easily read with the naked eye with the help of color‐based reporters.^15^

While many LAMP assays have been presented to detect norovirus by research groups, it is essential to systematically evaluate and conclude the performance of LAMP and the quality of these studies. So far, no overall analysis of the accuracy of LAMP for norovirus seems to have been researched. Therefore, here, we intend to determine the accuracy of LAMP methods for the diagnosis of norovirus infection, using systematic review and meta‐analysis techniques.

## 2 METHODS

### 2.1 Electronic searches

A systematic evaluation was conducted in this study according to the PRISMA guidelines.^16^, ^17^, ^18^ Two researchers independently retrieved relevant literature up to January 2021 by using the databases, including PubMed, Web of Science, Cochrane Library, Embase, CNKI (China National Knowledge Infrastructure), Wan Fang, and VIP. The first four were in English, and the next three were in Chinese. The primary keywords combined LAMP with norovirus. The search had no restriction in language, but synonymous extensions were utilized.

### 2.2 Study screening and selection

According to the predefined inclusion and exclusion criteria, two researchers (Xi‐Feng Qian and Nan‐Xi Li) as a group independently screened all English articles, including titles, abstracts, and full texts. Another group (Ai‐Ling Duan and Rong‐Xian Huang) were responsible for all Chinese articles in the same ways. In addition, we scanned the bibliographies of most publications included to identify additional studies. All of them used Endnote X9 to manage and screen the literature. Then, they checked and rechecked the results. A third researcher would join to achieve consensus if there was still a discrepancy after discussing in each group.

### 2.3 Inclusion and exclusion criteria

We established the inclusion and exclusion criteria by skimming several relevant works before screening the articles in detail. The inclusion criteria were listed as follows: (1) The study described LAMP and norovirus and linked them with the diagnostic tests; (2) the study was an original work of research; (3) RT‐PCR and LAMP had been used to detect the samples, and RT‐PCR had been used as the gold standard in the diagnosis of norovirus infection; and (4) there was enough information to extract 2*2 tables to form the pooled data. The criteria for exclusion were as follows: (1) duplicated publications, irrelevant studies, letters, erratum, reviews, and meeting abstract; (2) studies did not use the gold standard or could not extract the 2*2 data; and (3) the samples were collected from animals or other species.

### 2.4 Data extraction

While reading the included articles independently, data were extracted simultaneously on author, year, study design, country, detection method, gold standard, sample source, sample type, sample size, incubation temperature (°C) and time (min), detection limit, genogroup type, true positive (TP), false positive (FP), false negative (FN), and true negative (TN). The third researcher in the other group intervened to resolve the conflicts.

### 2.5 Quality assessment

We conducted the quality assessment in the included articles independently with the standard principles according to the Quality Assessment of Diagnostic Accuracy Study‐2 (QUADAS‐2) guidelines.^19^ It contained eleven criteria in four parts (patient selection, index test, reference standard, and flow and timing). The criteria of QUADAS‐2 are listed in Table S1. We responded with “Yes (Y), No (N), and Unclear (UC)” to assess the risk of bias and responded with “Low (L), High (H), and Unclear (UC)” in the concern of applicability. Then, the quality assessment form was filled with relevant data according to the included articles. We then discussed the result, and the third researcher resolved the dissents. Then, the statistical software Review Manager 5.3 was utilized to generate the quality plot.

### 2.6 Statistical analysis

Based on the data we extracted earlier, Meta‐DiSc 1.4 was employed to analyze the data and generate the results pertaining to sensitivity, specificity, negative and positive likelihood ratio (LR), diagnostic odds ratio (OR), and SROC.^20^ The pooled data were discussed to determine the accuracy of LAMP for the detection of norovirus. Heterogeneity analysis was discussed from the threshold and non‐threshold effects. The source of the heterogeneity was explored through the following operations: visual inspection of forest maps to observe the deviation and the inconsistency in the above‐combined results and analysis of correlation index^21^, ^22^; a large deviation and inconsistency between the studies indicated a possible source of heterogeneity. The random‐effects model was used to analyze the accuracy of the diagnostic method through presenting forest spots if large heterogeneity was found. Deeks’ funnel plot symmetry tests were conducted by Stata 15.0 to check the potential publication bias.^23^

## 3 RESULTS

### 3.1 Database search results

After the previous detailed database search, a total of 202 publications were initially retrieved. The number then decreased to 99 after excluding the duplicates. On this basis, we excluded 68 studies after screening the abstracts and titles. After a full‐text review, we excluded 27 articles. Ultimately, eight articles were identified for inclusion.^24^, ^25^, ^26^, ^27^, ^28^, ^29^, ^30^, ^31^ The reasons for specific exclusion are shown in Figure S1.

### 3.2 Characteristics of eligible studies

Based on our strategy of data extraction, eleven sets of data extracted from the eight studies were included for meta‐analysis. The detailed characteristics of the included studies are summarized in Table 1. All articles were prospective studies and published between 2008 and 2017. LAMP detected in GI or GII or both of genogroup of norovirus, the results were compared with RT‐PCR. Among them, all articles reported data from stool samples; one collected vomitus samples, and one collected the samples from the anal swab, peripheral water, and barreled water. A total of 1553 samples were used to evaluate the diagnostic performance of LAMP.

**TABLE 1:** Characteristics of the included studies (*n* = 11)

First author	Year	Study design	Country	Gold standard	Sample type	Incubation temperature (°C) and time (min)	Value of the positive judgment	Genogroup	Sample size	TP	FP	FN	TN
Fukuda^24^	2008	Prospective	Japan	RT‐PCR	Stool and vomitus	62, 90	Unclear	GI	212	18	0	1	193
Fukuda^24^	2008	Prospective	Japan	RT‐PCR	Stool and vomitus	62, 90	Unclear	GII	212	167	1^a^	5	39
Iturriza‐Gomara^25^	2008	Prospective	United Kingdom	RT‐PCR	Stool	Unclear	Positive sample of <10^4^ dilution	GI	505	15	0	3	487
Iturriza‐Gomara^25^	2008	Prospective	United Kingdom	RT‐PCR	Stool	Unclear	Positive sample of <10^4^ dilution	GII	510	338	0	9	163
Iturriza‐Gomara^25^	2008	Prospective	United Kingdom	RT‐PCR	Stool	Unclear	Positive sample of <10^4^ dilution	GI、GII	510	350	0	11	149
Zongfeng Chen^26^	2009	Prospective	China	RT‐PCR	Stool	62, 45	Positive sample of <10^6^ dilution	GI、GII	108	8	4	2	94
Jianming Luo^27^	2012	Prospective	China	RT‐PCR	Stool	65, 60	Positive sample of <5^6^ dilution /NoV RNA load of >10^3^ copies	GII	93	34	0	2	57
Jianming Luo^28^	2014	Prospective	China	RT‐PCR	Stool	65, 60	Positive sample of <5^6^ dilution	GII	190	55	0	3	132
Shaohua Zhang^29^	2014	Prospective	China	RT‐PCR	Stool	62, 70	Positive sample of <10^5^ dilution	GII	57	6	0	0	51
Suzuki^30^	2015	Prospective	Japan	rRT‐PCR^b^	Stool	Unclear	NoV load of >10^5^ copies/g stool	GI、GII	232	132	1	13	86
Zhen Tan^31^	2017	Prospective	China	RT‐PCR	Stool et al^c^	65, 120	NoV DNA marker >100 bp	GII	151	36	6	1	108

Abbreviations: FN, false negative; FP, false positive; TN, true negative; TP, true positive.

^a^ Positive for RT‐nested PCR.

^b^ Real‐time RT‐PCR.

^c^ Including stool, anal swab, peripheral water, and barreled water.

### 3.3 Quality assessment

**FIGURE 1:**
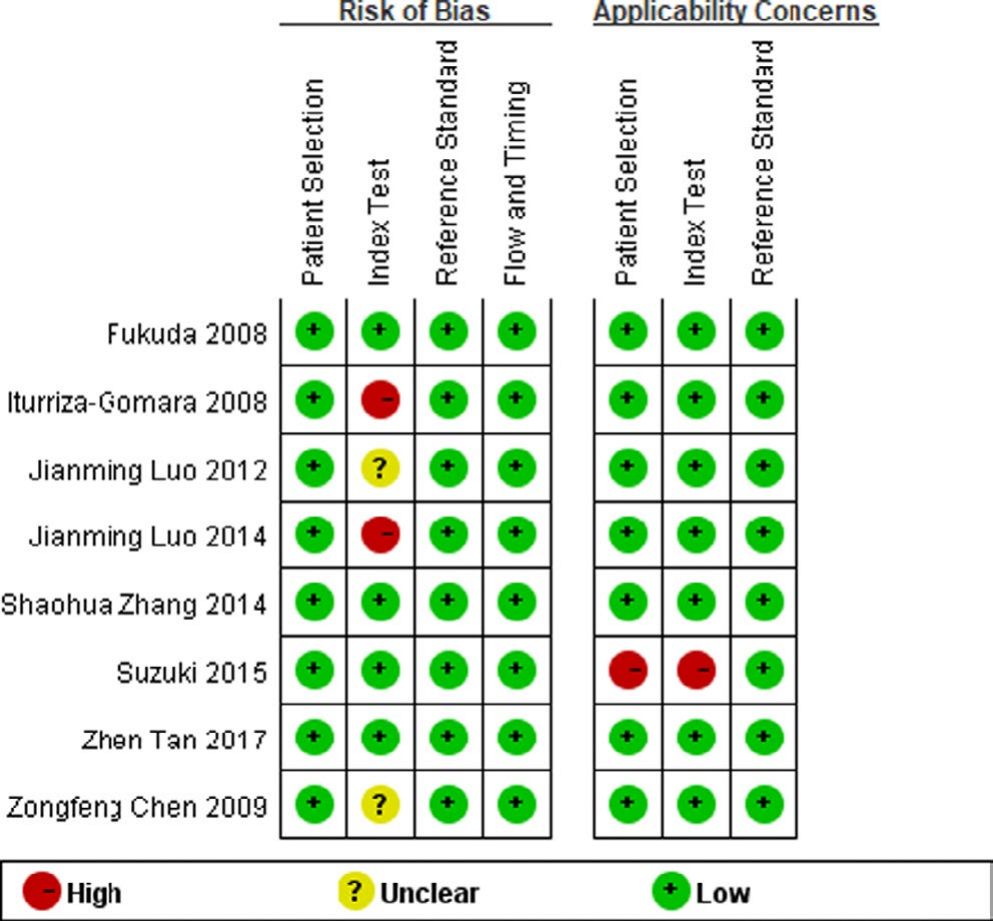
Quality evaluation of the included studies

**FIGURE 2:**
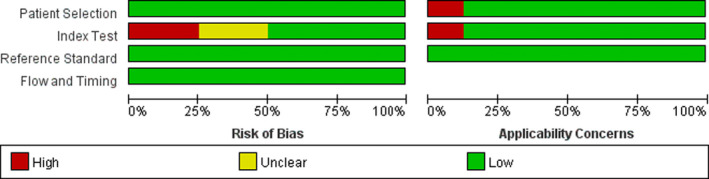
Risk of bias and applicability concern graph: review authors’ judgments about each domain presented as percentages across the included studies

To ensure scientific rigor and objectivity, two evaluations were conducted at different times. The quality assessment of the eight included articles is catalogued in Table S2. The overall risk of bias and applicability results are presented in Figures 1 and 2. Most studies were only performed when there were a low risk of bias and little concern about the applicability of the results. In the aspect of patient selection, all studies had a low risk of bias due to the inclusion of continuous or randomized samples and avoidance of inappropriate exclusions, while the applicability concerns were little high. In the aspect of the index test, approximately half of the studies were at low risk, while still two studies were classified as having “Unclear” risk of bias and one study as having a high risk of bias, because they were unclear in the elaboration of thresholds or explained the index test under the condition of knowing the results of the reference standards. In terms of the reference standard, all the studies had a low risk of bias and high clinical applicability, because the reference standard could correctly classify the target condition in all studies and the results of the reference standard were interpreted without knowledge of the results of the index test in more studies. In terms of flow and timing, a low risk of bias was existed in all studies, owing to that there was an appropriate interval between the index test and the reference standard and patients received the same reference standard. Also, all patients were included in the analysis except for the study of Iturriza‐Gomara et al.

### 3.4 Threshold effect analysis

The Spearman correlation coefficient is a nonparametric measure to evaluate the correlation between two statistical variables. If the Spearman correlation coefficient is more than 0.6, the possibility of threshold effect is indicated. Moreover, typical “shoulder‐arm” patterns indicate the presence of threshold effect.^20^ The Spearman correlation coefficient 0.164 with *p*‐value = 0.631 (*p* > 0.05) was applied, indicating the absence of a threshold effect, which would result in heterogeneity between the included studies. Moreover, as we observe in Figure 8, no “shoulder‐arm” distribution was seen. Thus, we concluded that no threshold effect existed among the articles included.

### 3.5 Non‐threshold effect analysis

Quantitative indicators of heterogeneity were judged by the inconsistency index (*I*‐square) automatically generated by Meta‐DiSc software. The inconsistency index was interpreted in the handbook of heterogeneity.^32^ The ratios are plotted with the forest map with a random pattern, and the result is shown in Figure 8 with the following values: Cochran's Q = 14.91, *p* = 0.1352 (*p* > 0.05), and inconsistency = 32.9% (inconsistency < 50%). This indicated that the heterogeneity originating from the non‐threshold effect was low.

### 3.6 Merge analysis results

**FIGURE 3:**
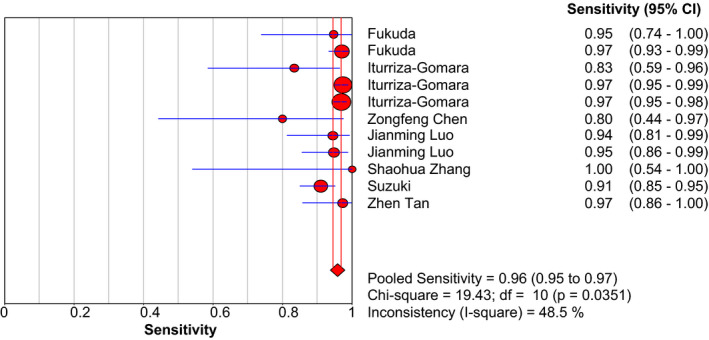
Forest plots for the combined sensitivity of LAMP

**FIGURE 4:**
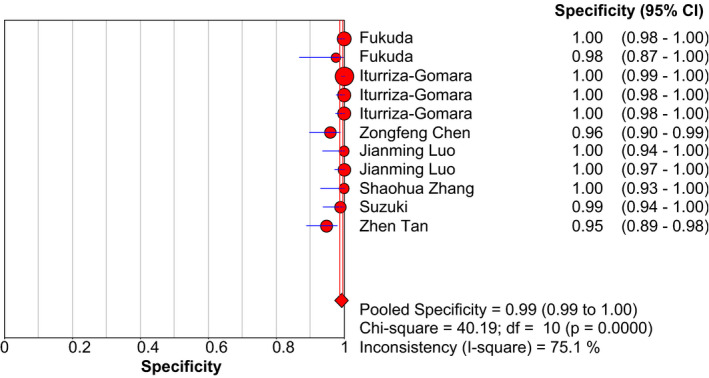
Forest plots for the combined specificity of LAMP

**FIGURE 5:**
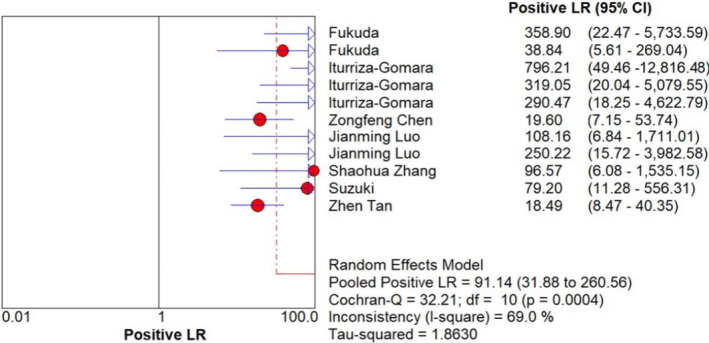
Forest plots for the combined positive LR of LAMP

**FIGURE 6:**
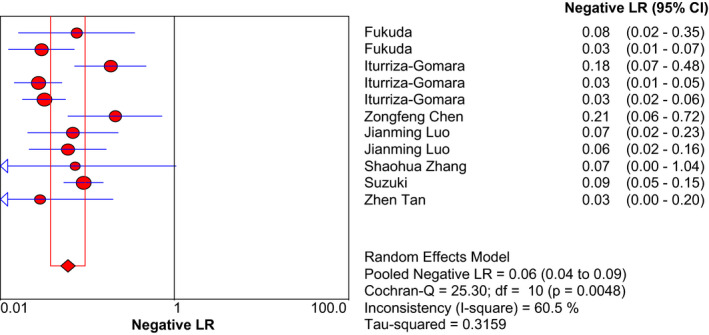
Forest plots for the combined negative LR of LAMP

**FIGURE 7:**
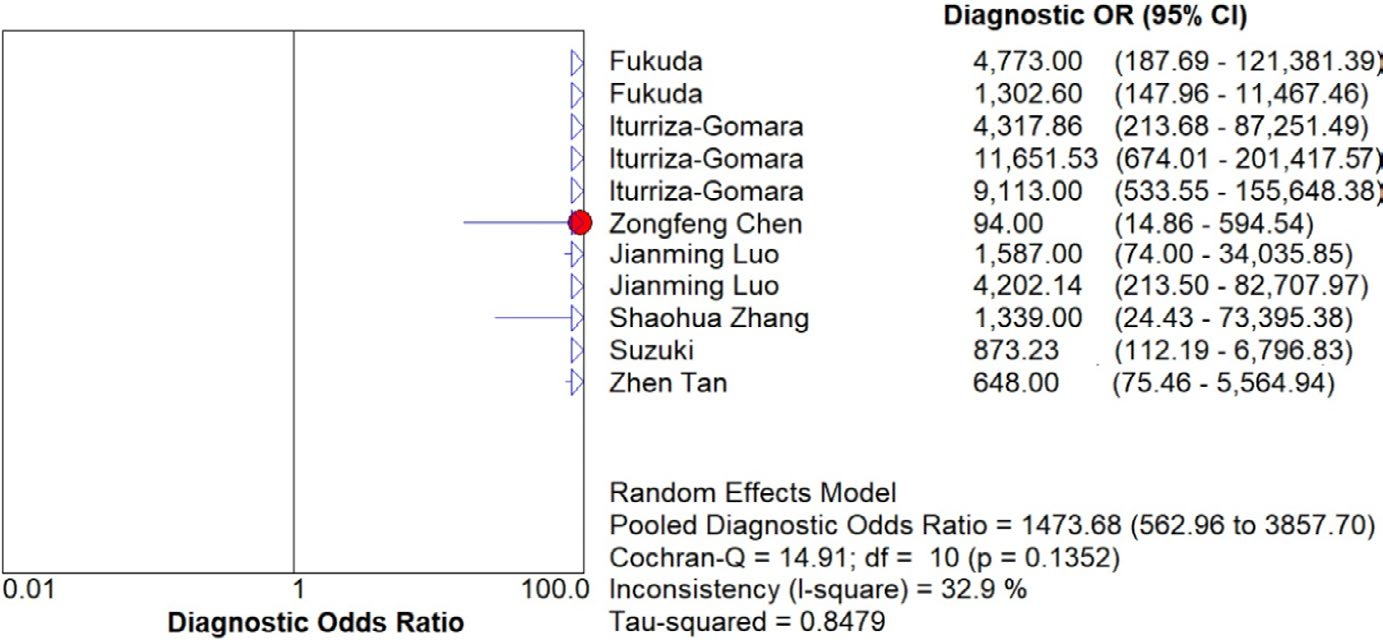
Forest plots for the combined diagnostic OR of LAMP

**FIGURE 8:**
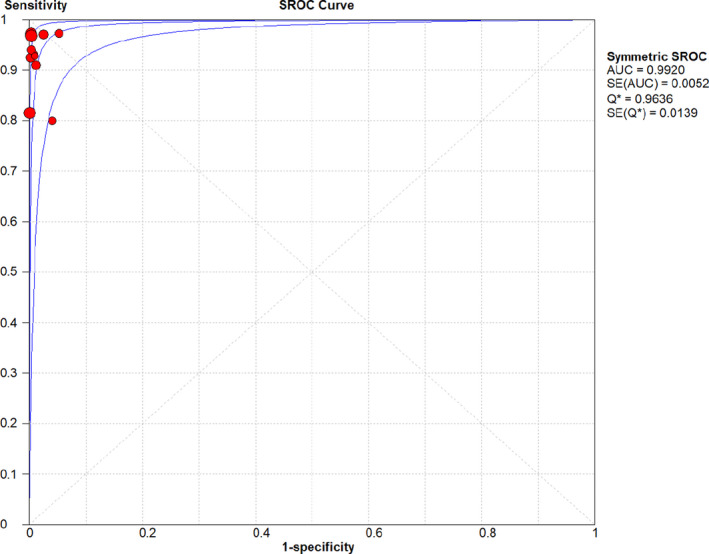
Summary receiver operating characteristic curves of norovirus infections detected by LAMP

With using the random‐effects model, the pooled data to evaluate the diagnostic accuracy of LAMP are shown in Figures 3, 4, 5, 6, 7. LAMP technology was utilized to detect norovirus, whose pooled sensitivity, specificity, positive LR, negative LR, and their 95% CI were 0.96 (0.95–0.97), 0.99 (0.99–1.00), 91.14 (31.88–260.56), 0.06 (0.04–0.09), and 1473.68 (562.96–3857.70), respectively. Moreover, the value of the combined diagnostic OR was 1473.68 (562.96–3857.70). Diagnostic OR is the ratio of positive LR to negative LR, reflecting the degree of connection between the results of diagnostic tests and diseases. The larger the diagnostic OR value, the better the discrimination effect of the diagnostic test. The SROC curve is shown in Figure 8, with AUC and Q* of 0.9920 and 0.9636 (SE = 0.0139), respectively. SROC is a comprehensive index reflecting continuous variables of sensitivity and specificity and revealing the relationship between them, and the AUC means the area under the SROC curve. The value of AUC getting closer to 1 indicates a better diagnosis, and Q* point is the intersection of the upper left and lower right diagonal with the SROC curve; the closer it is to the upper left, the better it is. According to the above results, it indicates that LAMP has high accuracy in the area of diagnosis of norovirus infection.

### 3.7 Publication bias

**FIGURE 9:**
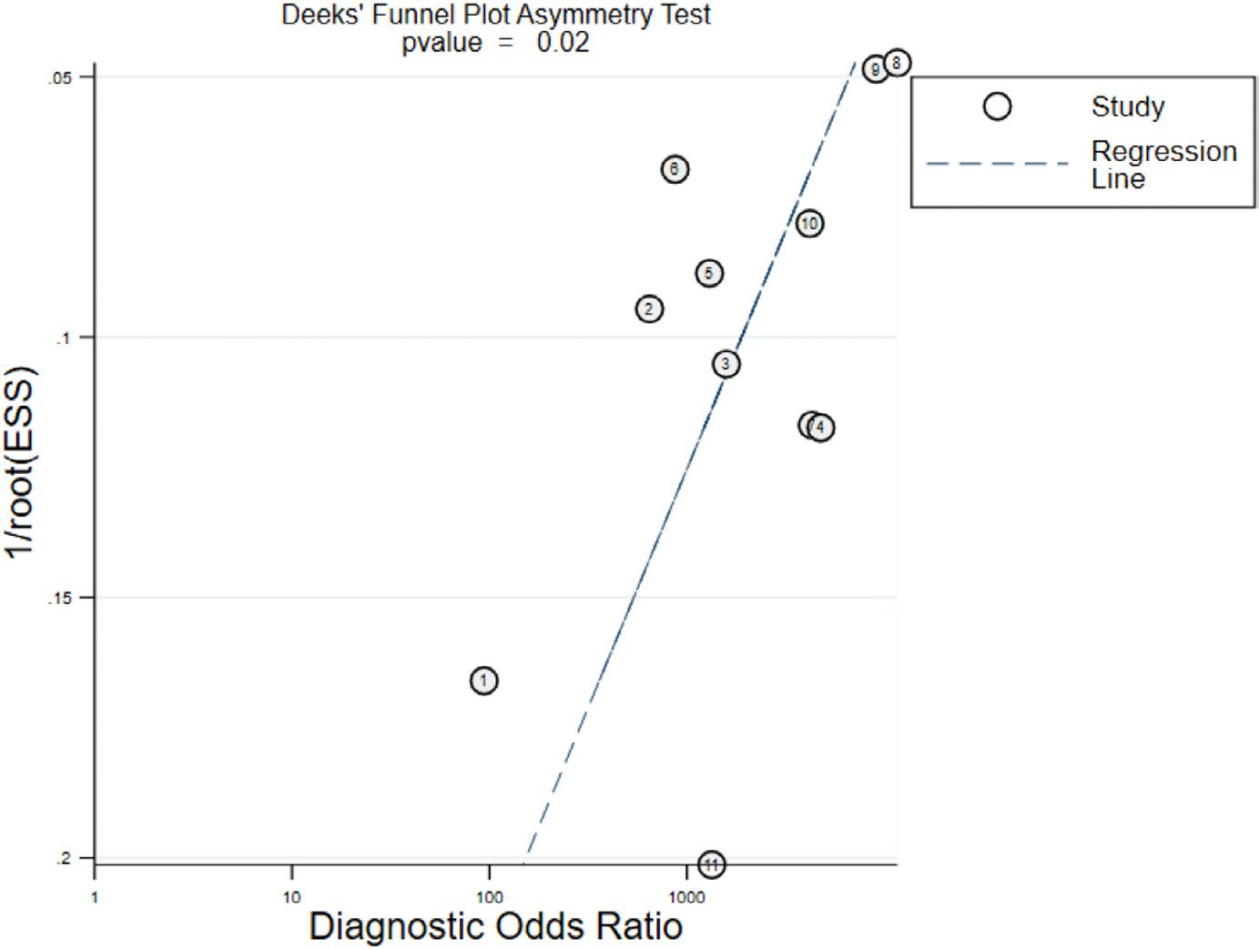
Deeks’ funnel plot asymmetry test to assess publication bias in estimates of diagnostic OR for LAMP detection of norovirus infections

Deeks’ funnel graph asymmetry was drawn, and the result, *p* = 0.02 (*p* < 0.05), is shown in Figure 9, which indicated a low potential for publication bias in the present study. However, the small number of studies included in the analysis resulted in a low capacity to detect bias.

## 4 DISCUSSION

Human norovirus is the leading cause of foodborne illness globally, which has caused a considerable public health and economic burden.^33^ Complex pathogenesis, different susceptibility, and difficult culture methods in vitro limit the research, diagnosis, and prevention of viruses.^33^, ^34^ A rapid and distinct diagnosis of norovirus infection is of great importance to treat patients early and control disease progression.

Among the detection methods of norovirus, RT‐PCR is regarded as the gold standard in the detection of norovirus with inherent advantages. However, the disadvantages of RT‐PCR–based assays lead to the limitation in its use in resource‐poor settings without equipped laboratories. It is usually only performed in public health laboratory with advanced research equipment.^35^

LAMP is a novel, rapid, specific, sensitive, and simple nucleic acid amplification technique located in six regions in the target gene using four primers and amplified RNA at a constant temperature. The most significant one is its rapidity; LAMP allows immediate diagnosis in just 30–50 min.^36^ Recently, LAMP has been utilized for the rapid detection of some pathogens, and their results showed high accuracy and availability in the diagnosis of the corresponding pathogens.^37^, ^38^, ^39^ In the detection of norovirus, LAMP with hydroxynaphthol blue dye could be observed visually through the change of color; therefore, the instrument of molecular electrophoresis is not required.^27^, ^28^, ^31^ This can decline costs, reduce procedure, and rapidly make a clear diagnosis. The previous study showed that the relative detection limitation was the concentration of the positive sample after 5^6^ dilution or norovirus RNA load of >10^3^ copies/tube, which was equivalent to that of the conventional RT‐PCR.^27^, ^28^ Therefore, LAMP has more advantages in detecting norovirus.

In this study, we majorly focused on the importance and applicability of LAMP in detecting norovirus promptly. Based on the strict inclusion and exclusion criteria, we finally included eight articles to form quality evaluation and extracted eleven data sets for meta‐analysis. The results in this analysis displayed that the pooled sensitivity and specificity of LAMP were 0.96 (0.95–0.97) and 0.99 (0.99–1.00), respectively, suggesting that LAMP had a lower rate of omission diagnosis and misdiagnosis in detecting norovirus. Furthermore, the negative LR, positive LR, and diagnostic OR were 0.06 (0.04–0.09), 91.14 (31.88–260.56), and 1473.68 (562.96–3857.70), respectively. A greater value contributed to a better distinguishing efficiency of diagnostic experiments if diagnostic OR was >1. Meanwhile, the AUC was 0.9920 and Q* was 0.9636 in SROC; the curve was close to the top left corner. The results proved a higher value, suggesting a greater diagnostic potential of LAMP for norovirus infection.

From the comprehensive analysis, one may conclude that the capacity of LAMP in the early diagnosis of norovirus is considerably high. LAMP may be a practical and reliable method for detecting norovirus.

In addition, Deeks’ funnel plots and the linear regression pattern were made to examine the publication bias of the included studies. The results implied the presence of publication bias, which might be that some negative results had not been published. It reduced the reliability and persuasiveness for the accuracy of LAMP of this analysis.

Further analysis was employed to investigate the source of heterogeneity of the included studies. To minimize the heterogeneity, this study had set rigorous inclusion/exclusion criteria earlier. The value of *I* ^2^ of pooled sensitivity (48.5%), specificity (75.1%), negative LR (60.5%), and positive LR (69.0%) indicated the existence of heterogeneity in this study. The threshold effect and non‐threshold effects were analyzed to discover the heterogeneous sources. The correlation index (0.164) and *p*‐value (0.631) of eleven data showed the lack of diagnostic threshold. Moreover, in non‐threshold effect analysis, the result of diagnostic OR (*I* ^2^: 32.9%) indicated the presence of non‐threshold effect in the included studies. Considering the few articles included, subgroup analysis was not conducted to investigate its heterogeneity. Through browsing the included articles, possible factors of heterogeneity originating from non‐threshold effect might be identified, including the virus conditions in patients, concurrent infection, the sample situation (collection, storage, and transportation), and experiment status (technics, standard tests, and operators).

Nevertheless, this analysis has several limitations. First, although we searched and screened all the relevant documents with the retrieval strategies, it was difficult to ensure that no articles were missing. Second, unpublished studies that might have several negative outcomes were not researched, which led to publication bias. Third, the number of the included articles was comparatively small. There were only eight articles to include and evaluate. Fourth, some articles included were relatively old. Half of the included articles were published before 2012. Finally, the existence of generic quality in the included studies could result in the rise of heterogeneity.

In conclusion, the study illustrates that LAMP has high sensitivity and specificity in the detection of norovirus. LAMP proves feasible and promising in the rapid diagnosis of norovirus infection. The results of this study provided information for finding an efficient method for the clinical detection of norovirus. However, further extension of this approach is encouraged to ensure the accuracy and practicability of this hopeful test in the future.

## CONFLICT OF INTEREST

The authors guarantee that there were no competing interests.

## AUTHOR CONTRIBUTIONS

XG and XF conceived and designed the experiments. XF and RX searched literature. XF, NX, AL, and RX screened literature, extracted data, and assessed the quality. XF contributed to the production of figures and tables by the analysis tools. YP was mainly responsible for the overall thinking. All authors participated in the writing, reading, and revising of the article and approved of the final version of the article.

## Supporting information

Figure S1

Table S1

Table S2

## Data Availability

All data generated or analyzed during this study are included in this published article.
