# Supplementary material for: Pooled analysis of LAMP assay for the diagnosis of norovirus infection
Source: J Clin Lab Anal. 2021 Jul 31;35(9):e23919. doi: 10.1002/jcla.23919 (PMC8418469; doi:10.1002/jcla.23919)
Supplement: Supplementary file 1 — Figure S1 [file JCLA-35-e23919-s001.doc]

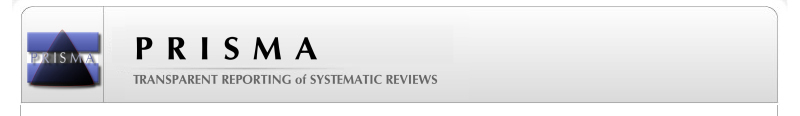
**PRISMA 2009 Flow Diagram**

**Screening**

**Included**

**Eligibility**

**Identification**

Records identified through database searching
(n = 202)

Additional records identified through other sources
(n = 0)

Records after duplicates removed
(n = 103)

Records screened
(n = 103)

Records excluded by screening title/abstract

（n=68）

16 Studies for other purpose

30 Irrelevant studies

10 Samples from other species

10 Reviews

1 Erratum

1 Letter

Full-text articles assessed for eligibility
(n = 35)

Full-text articles excluded, with reasons

(n=27)

13 Unable to form 2*2 table

7 Lack of reference standard

5 Reviews

2 Meeting Abstracts

Studies included in qualitative synthesis
(n = 8)

Studies included in quantitative synthesis (meta-analysis)
(n = 11)
