# Supplementary material for: Pooled analysis of LAMP assay for the diagnosis of norovirus infection
Source: J Clin Lab Anal. 2021 Jul 31;35(9):e23919. doi: 10.1002/jcla.23919 (PMC8418469; doi:10.1002/jcla.23919)
Supplement: Supplementary file 2 — Table S1 [file JCLA-35-e23919-s003.docx]

| Table S1 The criterions of QUADAS-2 to evaluate the quality | | | | | | | | | | | | | | | | | | | |
| --- | --- | --- | --- | --- | --- | --- | --- | --- | --- | --- | --- | --- | --- | --- | --- | --- | --- | --- | --- |
| Author | Year | Patients’ selection | | | | | Index test | | | | Reference standard | | | | Flow and timing | | | | |
|  |  | 1 | 2 | 3 | Risk of bias | Applicability concerns | 4 | 5 | Risk of bias | Applicability concerns | 6 | 7 | Risk of bias | Applicability concerns | 8 | 9 | 10 | 11 | Risk of bias |
|  |  |  |  |  |  |  |  |  |  |  |  |  |  |  |  |  |  |  |  |
|  |  |  |  |  |  |  |  |  |  |  |  |  |  |  |  |  |  |  |  |
|  |  |  |  |  |  |  |  |  |  |  |  |  |  |  |  |  |  |  |  |
|  |  |  |  |  |  |  |  |  |  |  |  |  |  |  |  |  |  |  |  |
|  |  |  |  |  |  |  |  |  |  |  |  |  |  |  |  |  |  |  |  |

1: Was a consecutive or random sample of patients enrolled? 2: Was a case-control design avoided? 3: Did the study avoid inappropriate exclusions? 4: Were the index test results interpreted without knowledge of the results of the reference standard? 5: If a threshold was used, was it pre-specified? 6: Is the reference standards likely to correctly classify the target condition? 7: Were the reference standard results interpreted without knowledge of the results of the index tests? 8: Was there an appropriate interval between index test and reference standard? 9: Did all patients receive the reference standard? 10: Did all patients receive the same reference standard? 11: Were all patients included in the analysis?
