# Supplementary material for: Pooled analysis of LAMP assay for the diagnosis of norovirus infection
Source: J Clin Lab Anal. 2021 Jul 31;35(9):e23919. doi: 10.1002/jcla.23919 (PMC8418469; doi:10.1002/jcla.23919)
Supplement: Supplementary file 3 — Table S2 [file JCLA-35-e23919-s002.docx]

| Table S2 Quality assessment of the included studies | | | | | | | | | | | | | | | | | | | |
| --- | --- | --- | --- | --- | --- | --- | --- | --- | --- | --- | --- | --- | --- | --- | --- | --- | --- | --- | --- |
| Author | Year | Patients’ selection | | | | | Index test | | | | Reference standard | | | | Flow and timing | | | | |
|  |  | 1 | 2 | 3 | Risk of bias | Applicability concerns | 4 | 5 | Risk of bias | Applicability concerns | 6 | 7 | Risk of bias | Applicability concerns | 8 | 9 | 10 | 11 | Risk of bias |
| Fukuda | 2008 | Y | Y | Y | L | L | Y | UC | L | L | Y | Y | L | L | Y | Y | Y | Y | L |
| Iturriza-Gomara | 2008 | Y | Y | N | L | L | N | UC | H | L | Y | Y | L | L | Y | Y | Y | N | L |
| Zongfeng Chen | 2009 | N | Y | Y | L | L | UC | UC | UC | L | Y | UC | L | L | Y | Y | Y | Y | L |
| Jianming Luo | 2012 | N | Y | Y | L | L | UC | UC | UC | L | Y | UC | L | L | Y | Y | Y | Y | L |
| Jianming Luo | 2014 | Y | Y | Y | L | L | N | Y | L | L | Y | Y | L | L | Y | Y | Y | Y | L |
| Shaohua Zhang | 2014 | Y | Y | Y | L | L | Y | Y | L | L | Y | Y | L | L | Y | Y | Y | Y | L |
| Suzuki | 2015 | Y | Y | Y | L | H | N | Y | L | H | Y | Y | L | L | Y | Y | Y | Y | L |
| Zhen Tan | 2017 | N | Y | Y | L | L | UC | Y | L | L | Y | UC | L | L | Y | Y | Y | Y | L |
